# Analysis of the unpredictable migration of impacted mandibular third molars: A pilot study

**DOI:** 10.4317/jced.56891

**Published:** 2020-12-01

**Authors:** Cintia-Micaela Chamorro-Petronacci, Mario Pérez-Sayáns, Cosme Gay-Escoda, Berta Rivas-Mundiña, Alejandro-Ismael Lorenzo-Pouso, Pilar Gándara-Vila, Arturo Bilbao-Alonso, Abel García-García

**Affiliations:** 1 DDS, PhD. Oral Medicine, Oral Surgery and Implantology Unit. Faculty of Medicine and Dentistry; Santiago de Compostela University. Health Research Institute of Santiago de Compostela (Instituto de Investigación Sanitaria de Santiago, IDIS), Santiago de Compostela, Spain; 2 MD, DDS, MS, PhD, EBOS, OMFS. Chairman and Professor of Oral and Maxillofacial Surgery and Implantology. Faculty of Medicine and Health Science. University of Barcelona. Director of the master’s degree programme in Oral Surgery and Implantology (EFHRE International University - FUCSO). Coordinator / Researcher of the IDIBELL Institute. Head of the Oral Surgery and Implantology and Maxillofacial Surgery Department of the Teknon Medical Centre. Barcelona, Spain; 3 DDS. Oral Medicine, Oral Surgery and Implantology Unit. Faculty of Medicine and Dentistry; Santiago de Compostela University. Health Research Institute of Santiago de Compostela (Instituto de Investigación Sanitaria de Santiago, IDIS), Santiago de Compostela, Spain; 4 MD, PhD. Maxillofacial Surgery Service, Santiago de Compostela Hospital Complex (CHUS), Galician Health Service (SERGAS); 5 MD, PhD. Oral Medicine, Oral Surgery and Implantology Unit. Faculty of Medicine and Dentistry; Santiago de Compostela University. Health Research Institute of Santiago de Compostela (Instituto de Investigación Sanitaria de Santiago, IDIS), Santiago de Compostela, Spain

## Abstract

**Background:**

Eruption of an impacted mandibular third molar (3MM) is often unpredictable. The objective of this study was to establish the radiographic parameters of migration in patients whose 3MMs evolved unpredictably.

**Material and Methods:**

This was a retrospective observational study. Patients with unusual 3MM migration (away from their physiological eruption position with changes in the longitudinal and horizontal axes) and with at least two panoramic radiographs were included. To evaluate the radiographic parameters, images were superimposed, using mandibular angle and ipsilateral condyle as references.

**Results:**

Of a total of 2851 patients, four were included in our study. The average age of the patients at the time of the second X-ray was 41.75 (SD=8.42) years. The mean follow-up period was 111 (SD=59.09) months. The migration was caudal in three of the 3MMs (75%) and cranial in one (25%).

**Conclusions:**

Unpredictable 3MM migration is rare, and occurs mostly in the vertical direction with an average angle of 12 degrees. None of these migrations were related to any type of lesion. Our results reveal that, due to its unpredictable behaviour, impacted wisdom teeth have to be periodically radiographically evaluated even if surgical extraction is not indicated.

** Key words:**Tooth migration, third molar, ectopic tooth.

## Introduction

The variation in the position of impacted mandibular third molars (3MMs) over time is a subject of concern to patients and dental professionals. Depending on the clinician’s criteria, the treatment approach is usually wait-and-see or preventive extraction. Factors such as the age of the patient, 3MM position or the prospect of intensive orthodontic treatment influence the decision. Various studies on 3MM extraction estimate that between 18 and 40% of asymptomatic wisdom teeth are extracted without associated pathology, and most clinicians decide to extract these teeth to prevent pericoronitis (1).

Most authors agree that the dietary changes in humans have led to a decrease in the physiological activity of the jaw bones. This decrease in functional activity also alters jaw growth, reducing the space available for complete dental eruption (2).

The prediction of impacted 3MM eruption is sometimes complicated. Numerous studies establish parameters that can be used to predict 3MM migration (towards eruption, impaction or semi-impaction). Systematic reviews on the predictability of the 3MM position over time suggest that more longitudinal studies are needed to help evaluate the changes in the 3MMs (3). The articles on 3MM migration always describe the movement mesially and, in some cases, towards the occlusal plane (3-11). Another described movement away from the occlusal plane, although not in 3MMs, is the transmigration of the canines moving to the contralateral maxillary side, passing the midline. Although it is possible to determine the cause of this phenomenon in some cases (e.g., follicular cyst, odontomas or supernumerary teeth), in other cases remain unknown (12).

The objective of our study was to describe the radiological parameters to predict or explain 3MM migration in an unusual direction, i.e., away from the longitudinal and horizontal axes of the adjacent second molar.

## Material and Methods

This was a retrospective observational study and was conducted according to the Strengthening the Reporting of Observational Studies in Epidemiology (STROBE) recommendations for observational studies (13). All procedures were carried out with the adequate understanding and written consent of the subjects in accordance with the Declaration of Helsinki.

The panoramic radiographs of all the patients in the Oral Medicine Unit of the School of Medicine and Odontology of the Universidad de Santiago de Compostela who underwent consultation for 3MM extraction were retrospectively reviewed.

The inclusion criteria were the following: patients with unusual 3MM migration, i.e., away from their physiological eruption position with changes in the longitudinal and horizontal axes of the adjacent second permanent molar; existence of at least two panoramic radiographs taken 5 years apart; access to the patient’s complete medical history; and patients who underwent anatomo-pathological study to rule out displacements caused by cystic lesions and/or tumours.

The exclusion criteria were the following: minors with 3MM impaction in a sTable position over time or with wisdom teeth in the mesioversion position or that had been moved by the absence of the adjacent second molar.

All the cases were evaluated by two independent investigators to determine patient inclusion. In case of disagreement, a third investigator acted as a mediator. All the parameters were measured by two independent researchers, and the measurements were accepted as valid when the differences between the evaluators’ measurements were less than 5%.

**Figure 1:**
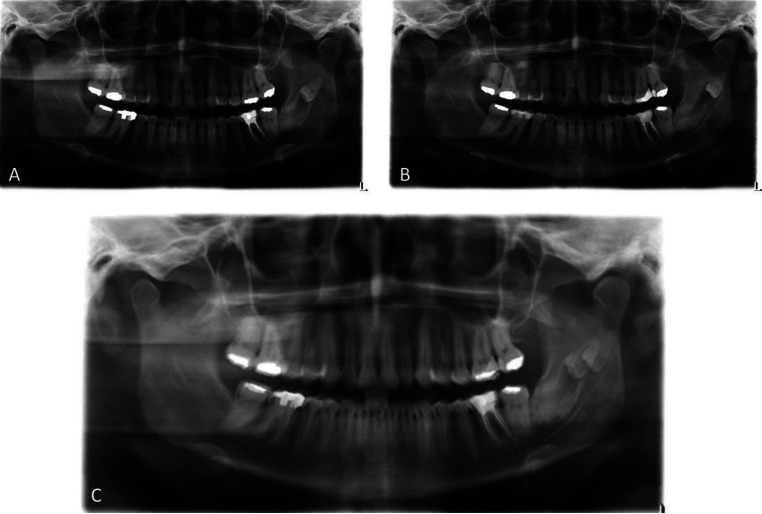
Example of two panoramic radiographs performed in 2009 (A) and in 2014 (B), and an example of the superposition (C) performed to analyse the migration over time of the third molar.

Taking into account that some of the panoramic radiographs were analogue and not digital, the images were superimposed with the image editing software Adobe Photoshop Creative Cloud 2014 (Adobe, Madrid, Spain), taking as reference the ipsilateral condyle, the mandibular angle and the duct of the inferior alveolar nerve (Fig. 1).

**Figure 2:**
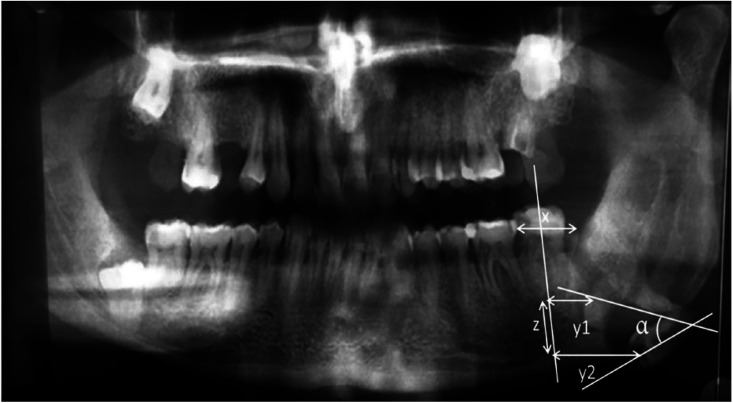
Radiographic measurements.

The mesio-distal distance of the second molar of the corresponding side in the first radiograph was used as the reference unit (X) in each patient. Horizontal measurements were taken from the longitudinal axis of the second molar by drawing lines perpendicular to the longitudinal axis of the wisdom tooth in the first X-ray (y1) and in the second X-ray (y2). The angle formed between the axes (y1 and y2) was also measured. The displacement on the 3MM horizontal axis was determined by the equation d=y2-y1. A third measurement (z) was used to assess the vertical displacement with respect to the longitudinal axis of the second molar, delimited by the points where both perpendicular lines (y1 and y2) intersected the longitudinal axis of the second molar (Fig. 2).

The variables collected were age, gender, side of 3MM, type of migration (distal, caudal, cranial), symptoms associated with 3MM, histopathological analysis, distance travelled along the horizontal axis, distance travelled along the vertical axis and time elapsed since the first and second X-rays (months).

All data were entered in duplicate into the database by two independent researchers to eliminate input errors. The analyses were carried out using the SPSS package, version 23.0 (SPSS, Inc., Chicago, USA).

The data were analysed using descriptive statistics in the form of frequencies and percentages for categorical variables and in the form of means and standard deviations or medians and interquartile ranges as appropriate. A comparison of the mean 3MM migration values was performed using a paired Student’s t-test. The relationship between horizontal and vertical migration was studied using the Spearman correlation coefficient. For the relationship between radiographic and clinical parameters, the Mann-Whitney U test was used, with a significance value of *p*<0.05.

## Results

**Figure 3:**
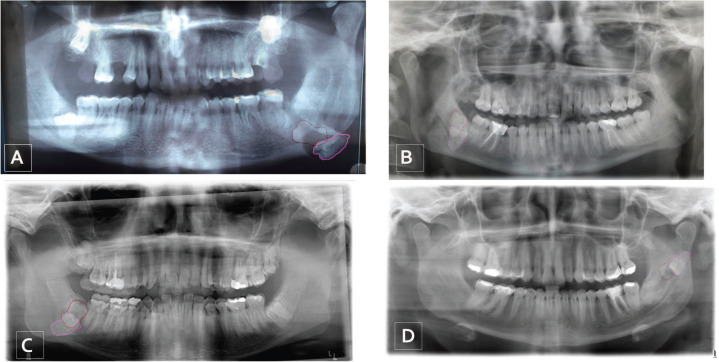
Patients included in the study and superposition with the initial position of the third molar outlined in red and the last position outlined in fuchsia; wisdom tooth A, 2001-2015; wisdom tooth B, 2012-2017; wisdom tooth C, 2005-2018; wisdom tooth D, 2009-2014.

A total of 2851 patient cases were reviewed from 1995 to 2018, and four patients (0.001%) fulfilled the inclusion requirements (Fig. 3).

The descriptive results of the study are summarized in Table 1. The average age of the patients at the time of the second X-ray was 41.75 (SD=8.42) years (age range 35-54). The patients presented no symptoms, with the exception of one patient who noticed a slight paraesthesia on the corresponding side. However, the 3MM movements were chance findings in the rest of the patients. No patient had received orthodontic treatment.

**Table 1:**
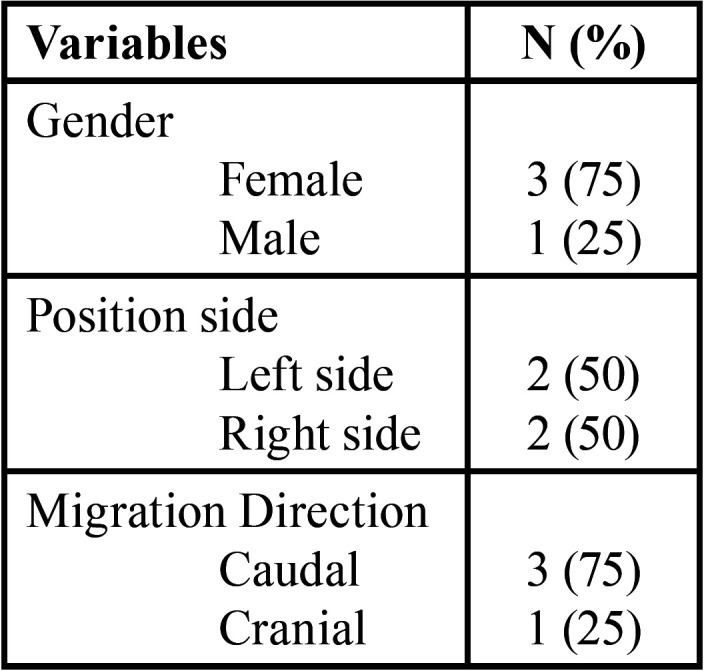
Descriptive Results.

Migration parameters are summarized in Table 2.

**Table 2:**
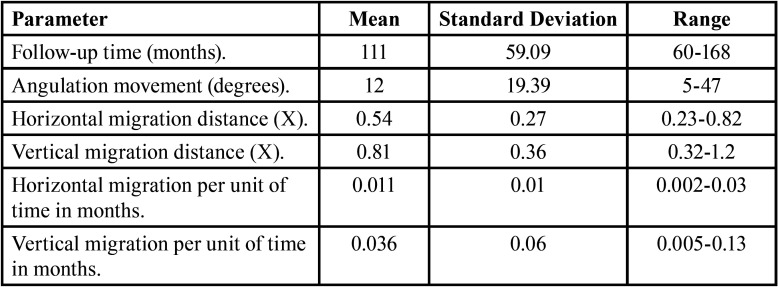
Migration Parameters. (X: mesio-distal distance of the second molar of the corresponding side in the first radiograph in each patient).

The Mann-Whitney U test did not reflect any relationship between wisdom tooth displacement and clinical variables, while we found a negative correlation between vertical migration and horizontal distance (CC=-0.949; *p*=0.05).

## Discussion

Four 3MMs were included in this study, and the mean follow-up time was 111 months. We were unable to relate any of the analysed variables to the average horizontal or vertical migration or the change in angulation over time. We found a negative correlation between the vertical migration and the horizontal distance (CC=-0.949; *p*=0.05), namely, as the vertical migration increases, the horizontal migration decreases.

Phillips and White (3) performed a bibliographic review with the intention of analysing longitudinal studies focused on the 3MM position changes. No study reported on the distalization or distancing of the third molar and the time of follow-up of the longitudinal studies varied (5,6,11,14), with the longest follow-up found in the study by Ventä *et al*. (18 years) (7).

All the 3MMs included in this study began their migration after the usual age of eruption, which, depending on the study, is between 17 and 21 years (11) or even during the third decade of life (4). Considering that the 3MM eruption peak usually occurs once jaw growth is complete, it does not appear that age can influence this unusual migration.

The studies that aim to predict 3MM eruption or impaction use their own software to digitally analyse the radiographic parameters. Different variables are measured in these cases, such as the distance from the distal axis of the second molar to the mandibular ascending ramus, the surface distal to Ricketts’s Xi point, angles formed by the 3MM axis and the gonion-symphyseal plane, angle between the second molar and the 3MM, angle between the mandibular ascending ramus and the mandibular plane (15). Other authors assessed parameters such as root resorption of the second molar, presence of periodontal disease and probing, presence of anterior crowding and the presence / absence of premolars (16,17).

One of the limitations of our study was the combination of digital and analogue radiographs, we were unable to use digital software to analyse the radiographic parameters since some of the radiographs were analogue. Other limitation is the number of patients, which we hope to increase in the future.

We did not find a relationship between the radiographic parameters studied and the clinical characteristics of the patient that explain the phenomenon.

Some authors have associated certain changes in the 3MM position with the presence or absence of periodontal disease (18). Although the patients in this study were not tested, patients showed no significant bone loss associated with this phenomenon of migration.

Obviously, this study did not aim to measure the actual 3MM migration distance but rather to evaluate this migration with the tools that we normally use to predict the 3MM position changes, i.e., panoramic radiograph. It should be noted that all patients had the second permanent molar on the migration side of the 3MM. In future measurements, other types of fixed points that are not a tooth should be used as references, as teeth are not always present in the mouth or migrate over time. However, it is possible that the presence of the final second molar helps explain the cause of this unusual migration.

The most common causes of movements such as transmigration are usually the presence of cysts (19), tumours (20), odontomas, supernumerary teeth, endocrine pathology or mandibular fractures (12). However, these causes were ruled out by our pathology department, as only fibro-connective tissue with non-specific chronic inflammatory changes in the biopsies performed at the time of the 3MM extraction surgery was found. While inflammation may initiate this process, we did not find evidence of inflammation in our patients that could explain the tooth migration. No patient took medication for chronic or inflammatory pathology.

The unpredicTable migration of the 3MMs included in this pilot study was not associated with any clinical or radiographic variable studied. To our knowledge, this is the first study that describes the unusual migration behaviour of impacted 3MMs that is unrelated to any pathological, bone or odontogenic lesion or a supernumerary tooth. Taking into account this new finding, we believe it is necessary to perform periodic panoramic radiographs, perhaps every two years, to assess 3MM movement and assess extraction in case the 3MM migration begins before the tooth can be extracted, which involves greater risk due to tooth movement to deeper bony planes. This is a preliminary study, and our group intends to continue increasing the study cohort to identify the causes that explain this unusual 3MM migration.
